# Systematic review of the costs and effectiveness of interventions to increase infant vaccination coverage in low- and middle-income countries

**DOI:** 10.1186/s12913-019-4468-4

**Published:** 2019-10-22

**Authors:** Cristina Munk, Allison Portnoy, Christian Suharlim, Emma Clarke-Deelder, Logan Brenzel, Stephen C. Resch, Nicolas A. Menzies

**Affiliations:** 1 Department of Global Health and Population, Harvard T.H. Chan School of Public Health, Boston, MA USA; 2 Center for Health Decision Science, Harvard T.H. Chan School of Public Health, Boston, MA USA; 3 Bill & Melinda Gates Foundation, Seattle, WA USA

## Abstract

**Background:**

In recent years, several large studies have assessed the costs of national infant immunization programs, and the results of these studies are used to support planning and budgeting in low- and middle-income countries. However, few studies have addressed the costs and cost-effectiveness of interventions to improve immunization coverage, despite this being a major focus of policy attention. Without this information, countries and international stakeholders have little objective evidence on the efficiency of competing interventions for improving coverage.

**Methods:**

We conducted a systematic literature review on the costs and cost-effectiveness of interventions to improve immunization coverage in low- and middle-income countries, including both published and unpublished reports. We evaluated the quality of included studies and extracted data on costs and incremental coverage. Where possible, we calculated incremental cost-effectiveness ratios (ICERs) to describe the efficiency of each intervention in increasing coverage.

**Results:**

A total of 14 out of 41 full text articles reviewed met criteria for inclusion in the final review. Interventions for increasing immunization coverage included demand generation, modified delivery approaches, cash transfer programs, health systems strengthening, and novel technology usage. We observed substantial heterogeneity in costing methods and incompleteness of cost and coverage reporting. Most studies reported increases in coverage following the interventions, with coverage increasing by an average of 23 percentage points post-intervention across studies. ICERs ranged from $0.66 to $161.95 per child vaccinated in 2017 USD. We did not conduct a meta-analysis given the small number of estimates and variety of interventions included.

**Conclusions:**

There is little quantitative evidence on the costs and cost-effectiveness of interventions for improving immunization coverage, despite this being a major objective for national immunization programs. Efforts to improve the level of costing evidence—such as by integrating cost analysis within implementation studies and trials of immunization scale up—could allow programs to better allocate resources for coverage improvement. Greater adoption of standardized cost reporting methods would also enable the synthesis and use of cost data.

**Electronic supplementary material:**

The online version of this article (10.1186/s12913-019-4468-4) contains supplementary material, which is available to authorized users.

## Background

A large body of evidence has demonstrated the effectiveness and cost-effectiveness of infant immunization for reducing the burden of vaccine-preventable diseases [1, 2]. Routine immunization programs, supplemented by periodic campaigns, cover the majority of the target population in most countries, yet in many settings coverage remains below program goals [3]. Expanding coverage is a major objective of immunization programs, both to increase the magnitude of health benefits from vaccination and to reduce disparities in outcomes among underserved populations [4]. The remaining gaps in target coverage are especially prominent in low- and middle-income countries (LMICs), and so these settings have been a focus for ongoing efforts to improve immunization efforts with both vertical and horizontal programs.

Several recent studies have been undertaken to describe the costs of providing national immunization services [5–12]. This research provides precise estimates of the cost of providing services at current coverage levels in a range of countries, and describes variation in the costs and operating practices of individual immunization sites. However, these studies do not provide direct evidence on the costs or cost-effectiveness of strategies and interventions used to scale up immunization coverage. Without this information, countries and the international stakeholders have little quantitative evidence on the best ways to use scarce resources to achieve immunization objectives. One possible source of information is studies that report the costs and effects of specific coverage-enhancing interventions.

We conducted a systematic review on the costs and effects of interventions to improve immunization coverage in LMICs, including research reported in the grey literature. This review updates past initiatives to survey the evidence on interventions to improve immunization coverage in low-income, high-burden settings [1, 2]. Several recent and historical studies have reviewed evidence related to immunization coverage improvements, but the majority of these have limited their scope to reporting effects on coverage or related programmatic outcomes, and excluded costs [13–19]. Three reviews have considered both costs and effectiveness. In 2004, Pegurri, et al. summarized evidence in the peer-reviewed literature [1] and Batt, et al. reviewed the relevant grey literature [2]. Both of these reviews identified a limited number of LMIC studies meeting inclusion criteria, and concluded that heterogeneity in methods adopted by these studies prevented quantitative synthesis of results. Both reviews found that few studies reported costs, with 10 out of 60 and 15 out of 34 identified studies including costs, respectively. A recent review by Ozawa, et al. including articles through 2016, adopted a broader scope, including peer-reviewed studies from both low- and high-income country settings, and covering all age groups, but excluding grey literature [20]. While this study was able to undertake some quantitative synthesis of results, the authors acknowledged difficulties due to the heterogeneity of methods and reporting adopted by included studies, and the majority of estimates in their final sample came from high-income settings.

Our review returns to the approach of the earlier reviews with a focus on LMIC settings. In our review, we extracted data for studies conducted after the period covered by the Pegurri, et al. and Batt, et al. reviews (prior to 2003), as earlier studies were already included in these prior reviews, and following the reasoning that older research would be less relevant to contemporary planning and budgeting decisions. These earlier reviews stressed the importance of the grey literature in documenting findings in this field, and consequently we included both the peer-reviewed and grey literature in our review [1, 2]. The objective of this review was to describe the incremental cost and effectiveness of interventions to increase coverage of infant immunization in LMICs, as defined by World Bank income group. The primary outcomes of interest were the incremental costs and incremental changes in target population coverage associated with a coverage-improvement intervention, as compared to routine program performance.

## Methods

This systematic review was registered with PROSPERO, an international prospective register of systematic reviews (record number 69586). We included interventions directed solely at increasing infant immunization coverage, as well as interventions designed to improve multiple aspects of immunization performance including coverage. The target age group for infants was defined as age 1 year and below; therefore, measles, mumps, rubella, and varicella vaccination interventions were included. We only included studies that reported empirical data, and excluded modeled analyses to minimize the introduction of additional bias into any summary results. We excluded interventions designed to improve health service delivery generally (e.g., improvements in access to primary health care). We also excluded studies that reported changes in immunization coverage but did not describe specific interventions used to impact infant immunization coverage. Studies with no data on intervention costs were excluded, as were those targeting adult immunization and animal studies. We included studies published between January 2003 (the end date of the period covered by older reviews [1, 2]) and May 2019.

### Search strategy and extraction

We identified eligible studies in the published literature by searching the following electronic databases, without language limitations: CEA Registry, Cochrane Library, EconLit, Embase, PubMed, Social Science Research Network (SSRN), and Web of Science [21–23]. We also reviewed the reference lists of recent reviews of coverage improvement interventions [13, 20] as well as selected studies found in the initial title search [24–30].

We identified eligible studies in the grey literature by searching relevant databases and repositories, including World Health Organization (WHO) regional databases Literatura Latino-Americana e do Caribe em Ciências da Saúde (LILACS) and African Index Medicus (AIM), ELDIS, the World Bank working papers, GreyNet, and Grey Literature Report [31]. Reports found to have a matching publication in the published literature were excluded.

We adopted previously developed sensitivity- and specificity-optimized search strategies for identifying healthcare cost studies and economic evaluations [31–34]. We tested the robustness of our search strategy by applying it to the timeframe of the Pegurri, et al. review in PubMed and confirming that all studies included in the earlier review were identified by our search terms. Additional file 1 lists the complete search terms utilized for each of the databases. Search terms fell into four categories: (1) immunization terms; (2) coverage terms; (3) cost terms; and (4) LMIC terms. Some databases limited the number of terms that could be searched; in such cases, the number of search terms was consolidated and, when necessary, distinct searches were performed for each category. Due to these limitations, LMIC search terms could not be included in every database.

Record titles and abstracts were independently screened by one of two reviewers (CM and AP) to identify studies that met the inclusion criteria. Reports not meeting study inclusion criteria were excluded. Uncertainty about study inclusion was resolved by a third independent investigator (NM).

From each of the included studies, we extracted information on the type of vaccination coverage intervention and study design; urban vs. rural setting; campaign vs. routine delivery; vaccination delivery platform; the baseline and endline coverage or incremental coverage, where applicable; the intervention costs and intervention cost per person exposed; and the incremental cost-effectiveness ratio. This information was extracted for each unique intervention or country in a study. For example, if a study analyzed two different interventions in two different countries, we extracted information related to each of the four observations.

### Analysis

#### Quality evaluation

We evaluated the methodological quality and risk of bias of included studies using the Consensus on Health Economic Criteria (CHEC) list [35]. We excluded the CHEC list items that were specific to modeling analyses, as our review focused on empirical analyses. For each of the relevant items on the checklist, we assigned studies a score of zero or one (Additional file 2) [35]. A score of “0” indicated that the selected element was not present in the article; “1” indicated the element was present.

#### Calculating incremental coverage

The primary outcomes of interest were the incremental costs and incremental changes in target population coverage associated with a coverage-improvement intervention, as compared to routine program performance. We calculated incremental coverage (defined as the percentage point change in vaccination coverage for the target group) based on the reported study design and outcomes. For studies that measured coverage at baseline and at the intervention conclusion, in both control (*ctl*) and intervention (*int*) groups (pre-post with control, including randomized control trials), incremental coverage was calculated as:

$$ Incremental\ coverage=\left({Intervention\ coverage}_{int}-{Baseline\ coverage}_{int}\right)-\left({Intervention\ coverage}_{ctl}-{Baseline\ coverage}_{ctl}\right). $$

In studies which measured coverage only at the intervention conclusion (post-test with control), incremental coverage was calculated as:

$$ Incremental\ coverage=\left({Intervention\ coverage}_{int}-{Intervention\ coverage}_{ctl}\right) $$

In studies that measured coverage before and after the intervention without a control group (pre-post without control), incremental coverage was calculated as:

$$ Incremental\ coverage=\left({Intervention\ coverage}_{int}-{Baseline\ coverage}_{int}\right) $$

#### Calculating incremental intervention cost

For studies that measured costs at baseline and at the intervention conclusion, incremental intervention costs were calculated as:

$$ Incremental\ intervention\ cost=\left( Intervention\ cost- Baseline\ cost\right) $$

If studies included only incremental intervention costs, with no baseline costs stated, the stated intervention costs were listed. All costs were converted to 2017 USD using local inflation according to the consumer price index and local currency to USD exchange rates [36, 37].

#### Calculating incremental cost effectiveness ratios (ICERs)

ICERs were calculated as:

$$ ICER= Incremental\ intervention\ costs/\left( Incremental\ coverage\ast Population\ exposed\ to\ intervention\right) $$

Some studies evaluated interventions that combined multiple health services, some of which were not focused on immunization [38, 39]. The costs reported by these studies were not broken out by immunization activities vs. other areas. For these studies, the ICER assumes that the costs of all included interventions are attributable solely to increasing immunization coverage, while the incremental coverage benefit is specific to immunization.

In other studies, the currency years of costs were unclear. We contacted the authors of these studies to request clarification of cost currency years, where possible, and assumed a currency year according to the year of the intervention otherwise. Nevertheless, several studies remained in which the ICER could not be calculated with the cost and coverage data provided.

## Results

**Fig. 1:**
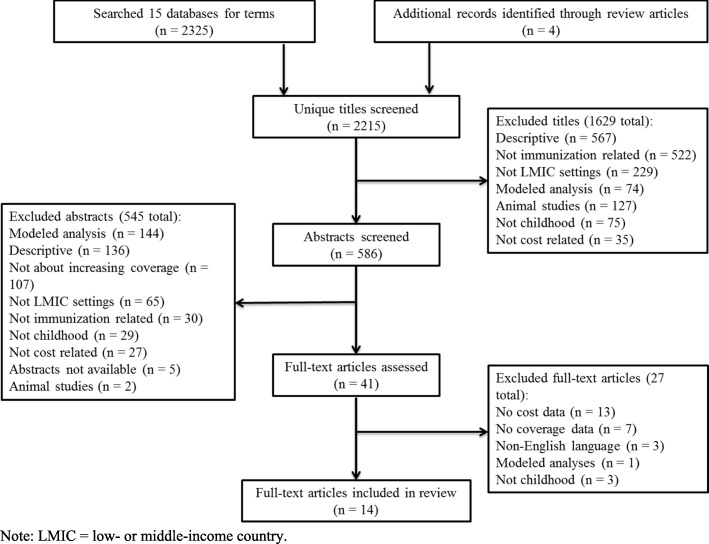
Flow diagram of articles included in the review

A total of 2325 records were identified from 15 databases (Fig. 1), with an additional 4 records obtained from reference lists of other articles. After removing 114 duplicates, 2215 titles were screened for eligibility, of which 1629 were removed. Of the 586 records remaining, 545 were removed after abstract review, leaving 41 articles which received full-text review. Of these, 27 were excluded for not meeting study inclusion criteria. We examined the LMIC studies included in the Ozawa review and added two relevant articles that we had previously excluded [40, 41]. Fourteen studies were included in the final analysis [38–51].

Table 1 describes general characteristics of the 14 studies. One study describes interventions from two countries (Mexico and Nicaragua); these were considered separate observations when extracting cost and effectiveness data [44]. More than half of the observations (*n* = 10) were conducted in Asia, with the remaining in Africa (Ethiopia, Guinea-Bissau, and Madagascar) and Central America (Mexico and Nicaragua). Observations were nearly evenly split with regard to setting: 6 were conducted in rural areas; 3 in urban; 3 in mixed settings and 3 were not specified. About half of the interventions (*n* = 7) were delivered in a mobile outreach format; 4 were delivered in a fixed (i.e., health facility) format, and 4 interventions utilized both a mobile and fixed format. Additionally, 10 interventions were administered within routine vaccination systems, while 5 were administered using a campaign format. One study utilized both routine and campaign formats. Intervention duration ranged from 2 weeks to 5 years.

**Table 1:** General features of the interventions

Publication Year	First author	Location	Study type	Intervention description	Intervention type	Urban / rural	Campaign vs. routine	Delivery platform^a^
2009	Andersson	Pakistan (Balochistan province)	Cluster randomized controlled trial	Community discussion groups on vaccine benefits, costs, and coverage	Demand generation	Not stated	Routine	Mobile
2010	Banerjee	India (Rajasthan state)	Cluster randomized controlled trial	Monthly immunization camps conducted by mobile team in villages	Delivery approach	Rural	Routine	Mobile
2007	Barham	Mexico (7 states)	Cluster randomized controlled trial	Cash transfers conditional on children attending preventative health visits and mothers attending health education talks	Demand generation / Cash transfers	Both	Routine	Both
		Nicaragua		Cash transfers conditional on children attending preventative health visits and mothers attending health education talks	Demand generation / Cash transfers	Not stated	Routine	Both
2017	Byberg	Guinea-Bissau (9 regions)	Cluster randomized controlled trial	Giving measles vaccination to all unvaccinated children 9–36 months regardless of number of children present	Delivery approach	Rural	Campaign	Mobile
2014	Carnell	Ethiopia (Amhara, Oromia and SNNP regions)	Pre-post design	I: Strengthen health systems (planning, HMIS, logistics, health care financing) II: Improve health workers’ skills (through training and supervision in immunization, ENA and IMCI) III: Introduce community health promoters	Health systems strengthening	Rural	Routine	Fixed
2003	Drain	Madagascar (Antananarivo and Fianarantsoa provinces)	Randomized controlled trial	Clinic staff used auto-disable syringes on all days or on non-routine immunization days	Novel technology	Both	Both	Fixed
2014	Hayford	Bangladesh (Dhaka)	Pre-post design	I: Extended hours at satellite clinics; II: training for vaccinators; III: clinic screening tool to identify children with missed doses; IIII: volunteer community group to assist at satellite clinics	Delivery approach	Urban	Campaign	Mobile
2013	Khan	Bangladesh (Mirpur area of Dhaka)	Cluster randomized controlled trial	Oral cholera vaccination for high-risk, urban population aged one and older	Delivery approach	Urban	Campaign	Both
2005	Levin	Indonesia (West Nusa Tenggara province)	Pre-post design	Delivering birth dose of Hepatitis B vaccine using prefilled injection device	Novel technology	Not stated	Routine	Mobile
2011	Owais	Pakistan (Karachi)	Randomized controlled trial	Home-based vaccine promotion education by community health workers using pictoral cards	Demand generation	Urban	Routine	Mobile
2007	Pandey	India (Uttar Pradesh state)	Cluster randomized controlled trial	4–6 meetings in each village to disseminate information on entitled health and education services	Demand generation	Rural	Routine	Mobile
2018	Powell-Jackson	India (Uttar Pradesh state)	Randomized controlled trial	Health information messaging targeting mothers of unvaccinated or incompletely vaccinated children through home visits	Demand generation	Rural	Routine	Fixed
2009	Rainey	India (Uttar Pradesh state)	Pre-post design	Identifying and vaccinating newborns with OPV within 72 h of birth	Delivery approach	Both	Campaign	Both
2006	Soeung	Cambodia	Cross-sectional design	Developing and implementing immunization microplans that are supported by performance based agreements and a secure system of financing	Health systems strengthening	Rural	Routine	Fixed

^a^‘Fixed’ refers to vaccinations delivered in a health facility; ‘mobile’ refers to vaccinations delivered through mobile outreach services

**Fig. 2:**
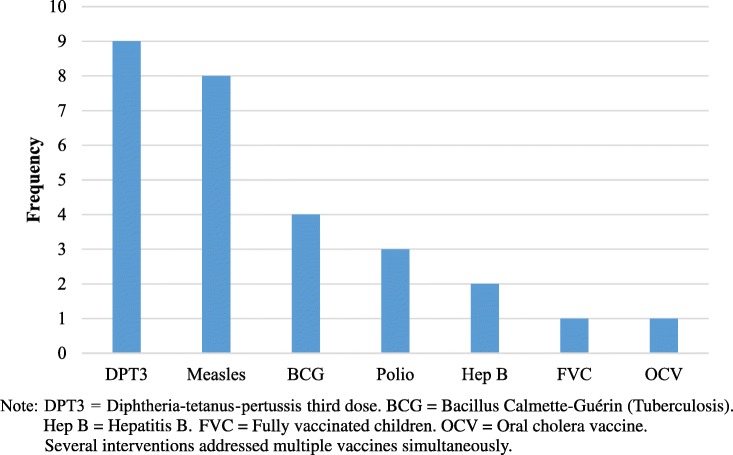
Vaccines interventions addressed

More than half of the studies (*n* = 9) reported randomized controlled trials, with the remaining studies reporting non-randomized pre-post evaluations (1 with control group, 3 without control groups) or cross-sectional designs. The majority of interventions were aimed at approaches for vaccine delivery (*n* = 5) or approaches to encourage additional vaccine uptake (i.e., demand generation; n = 5). Additional studies were aimed at health systems strengthening or the introduction of novel vaccine technologies (e.g., syringes). The interventions targeted coverage improvements for a range of vaccines, with DPT3 (diphtheria-pertussis-tetanus vaccine third dose) and measles vaccination most commonly addressed (Fig. 2).

### Coverage and cost information

Table 2 summarizes coverage and cost data in the reviewed studies. If relevant values were not provided directly by studies, values were derived from the study data, such as incremental coverage, intervention cost (converted to 2017 USD), intervention cost in 2017 USD per person exposed, and ICERs. Intervention and baseline costs were primarily presented as total costs, with some studies presenting cost per person exposed [50] or cost per community health worker trained [49].

**Table 2:** Coverage and cost characteristics of included studies

Publication Year	First author	Vaccine / intervention breakout	Baseline coverage	Endline coverage	Incremental coverage	Intervention cost (2017 USD)	Intervention cost per person exposed (2017 USD)	ICER
2009	Andersson	Measles			22%	$86,968	$162.25	$124.86
		DPT3			23%			$119.43
2010	Banerjee	Intervention A	2%	18%	11%	$41,109	$83.70	$1.09
		Intervention B	0%	39%	34%	$66,460	$41.89	$0.66
		Control	1%	6%				
2007	Barham^a^	Mexico: MCV treatment areas	92%	91%	3%^b^	$2303 million	$44.07	*^c^
		Mexico: MCV control areas	95%	91%				
		Nicaragua: FVC treatment areas	54%	83%	11%	$5,007,901	$67.11	*^d^
		Nicaragua: FVC control areas	55%	73%				
2017	Byberg		84%	97%	13%	$76,994^e^	$1.41	$3.29
2014	Carnell^f^	DPT3	45%	65%	8%	$26,049,434^g^	*^h^	*
		Measles	46%	64%	13%			
2003	Drain	Auto-disable syringes			16%	Not stated		$78.06
		Mixed syringes			17%			$5.03
2014	Hayford		43%	99%	56%	$36,190		$41.40
2013	Khan				72%	$680,581	$3.94	$5.50
2005	Levin		68%	80%	12%	$11,709^i^	$0.12	$1.00
2011	Owais	Intervention	77%	72%	19%	$1.15		*^j^
		Control	76%	52%				
2007	Pandey	Intervention	53%	72%	20%	$5997	$1.38	$6.88
		Control	47%	46%				
2018	Powell-Jackson	Intervention	0%	43%	15%	$11,137	$23.64^k^	$161.95^l^
		Control	0%	28%				
2009	Rainey		38%	65%	27%	Not stated	$3.72	$9.01
2006	Soeung				16%	$186,031	$2.20	$13.75

*DPT3* diphtheria-pertussis-tetanus vaccination third dose, *MCV* measles-containing vaccine, *FVC* fully-vaccinated children, *ICER* incremental cost-effectiveness ratio

*Data required to calculate ICER not included in study

^a^Selected interventions listed here; full results can be found in the paper

^b^Endline levels lower than baseline levels for both treatment and control areas, but the “program did lead to an equalization of vaccination rates between the treatment and control group, despite the treatment group’s coverage rate being 3 percentage points lower than in the control area at baseline” [44]

^c^Intervention contained a package of health services (immunization and other health activities). However, intervention costs were given only at an aggregate level and therefore immunization ICERs could not be calculated

^d^Intervention contained a package of health services (immunization and other health activities). However, intervention costs were given only at an aggregate level and therefore immunization ICERs could not be calculated

^e^Intervention cost minus hospital cost savings (both in USD 2017)

^f^Baseline and endline coverage estimates compiled from aggregating values for children 12–23 months in 6 intervention / control areas

^g^Includes both vaccination and non-vaccination interventions

^h^Size of the population exposed to the intervention not stated

^i^Only one province (of three described in study) was scaling up pre-existing immunization services. However, costs were reported as an annual net cost to the government for the new device across all three provinces. We conservatively assumed this aggregate cost was specific to the single relevant province for the cost per person exposed and ICER calculations

^j^Costs were only reported per community health worker. Costs per exposed child could not be determined

^k^Cost per mother given the information intervention

^l^Cost per additional child vaccinated with DPT3

Most studies reported increases in vaccination coverage following the interventions. In our review coverage increased by an average of 23 percentage points post-intervention across the studies, with rates ranging from 8 [38] to 72 [40] percentage points. Interventions aimed at improving and increasing delivery mechanisms saw the largest incremental coverage increases, at an average of 36 percentage points post-intervention [40, 43, 45, 47, 50]. While some studies did not provide baseline coverage, there was no apparent relationship between incremental coverage and baseline coverage, even when looking across intervention types. There was no discernable pattern between low-income and middle-income country studies.

Given the small total number and methodological heterogeneity of included studies, we decided not to attempt a quantitative synthesis of results. For those studies reporting coverage improvements with the intervention, ICERs ranged from $0.66 [43] to $161.95 [51] per child vaccinated in 2017 USD. There was also variation in the intervention costs reported. Studies most commonly reported site-level immunization costs (46%, *n* = 6). The next most common reported costs were supply chain and management costs (38%, *n* = 5), other costs (36%, n = 5), and vaccine costs (31%, *n* = 4).

### Quality ratings

Additional file 2 lists the quality scores each study received, according to the CHEC list, out of a total of 17 possible points. Scores ranged from 12 to 17, with a mean score of 14. Studies with the highest overall scores (averaging greater than or equal to 14 out of 17) were more likely to include appropriate costs and outcomes, as well as incremental analysis. Seven out of 14 included studies were missing items required for the incremental analysis that we conducted, indicating a risk of bias within the calculated ICERs.

## Discussion

We reviewed the recent literature describing the incremental cost and impact of efforts to improve immunization program coverage in LMIC, and identified 14 studies that containing sufficient cost and coverage data to be included in this study. The interventions reviewed in these studies covered a wide range of geographic settings, vaccines, intervention types, delivery mechanisms and scales. About half of the studies were randomized controlled trials, and the average study score on the CHEC list was 14 out of 17. The majority of included studies reported increases in vaccination coverage following the examined interventions, similar to the results of the Pegurri, et al. 2004 and Batt, et al. 2004 reviews [1, 2], which found coverage improvements of 27 percentage points and 20 percentage points on average, respectively.

Although all reviewed studies provided costs of the intervention itself, only 2 studies also considered changes in the costs of providing routine immunization services, despite the fact that such changes are a likely consequence of efforts to increase coverage. Eleven studies (79%) provided adequate data to calculate ICERs. From these, 14 ICERs of different interventions were calculated, ranging from $0.66 [43] to $161.95 [51] per child vaccinated in 2017 USD. In several cases, calculations of ICERs relied on substantial assumptions. Given the considerable differences in settings and perspectives, as well as different methods and cost categories included, an observed difference between ICERs may well reflect an artifact of the different study designs rather than a true feature of the interventions under study. Therefore, estimates should be compared with caution, and with full knowledge of the methodological and contextual differences between two studies. Funding devoted to coverage improvements may not be utilized efficiently in the absence of better evidence on optimal approaches.

Several factors prevented ICERs from being calculated in all studies. The interventions described in some studies [38, 44] contained a package of health services (immunization and other health activities). However, intervention costs were given only at an aggregate level and therefore immunization ICERs could not be calculated. Additionally, in one study the size of the population exposed to the intervention was not stated [38]. Similarly, in one study the costs were calculated at the level of health workers, so cost per exposed child could not be determined [49]. Due to the small total number and methodological heterogeneity of the extracted studies, we were unable to conduct a quantitative synthesis with cost estimates stratified by intervention type and country category.

Despite the 15-year gap since previous systematic reviews focusing on LMICs, there is still a scarcity of evidence on the cost-effectiveness of options for improving immunization coverage [1, 2, 20]. Studies rarely report estimates of the incremental change in both cost and coverage. Several of the studies that do provide such information failed to report key features of the study methods, which limits the utility of their results. In particular, future studies should include detailed discussions of the intervention type (i.e., aimed at demand generation, delivery mechanisms, etc.), target population, baseline and endline coverage, specific intervention costs, changes in costs of routine service provision as well as information on effect modifiers that could affect incremental costs and coverage. Such information should be reported at a level of granularity that other programs would be able to interpret and modify those costs to fit their individual setting. These details are necessary to navigate the heterogeneity of the studies and to directly compare and synthesize the results to produce generalizable conclusions. The biggest challenge is that efforts to scale up immunization coverage often lack provision for a costing study, which represents a huge missed opportunity to understand the best use of scarce resources for improving coverage. We challenge the immunization community at country, regional, and global levels to incorporate costing studies into scaling up efforts.

## Conclusions

This research underscores the need for broad adoption of standardized reporting methods in immunization costing studies [52, 53]. This will be essential to increase our knowledge of improving immunization coverage as well as child immunization intervention costs.

## Additional files

Additional file 1: Search terms for each database. (DOCX 16 kb)

Additional file 2: Consensus on Health Economic Criteria (CHEC) list for health economic evaluations^35^ and study scores. (DOCX 19 kb)

## Data Availability

The dataset supporting the conclusions of this article is included within the article (and its additional files).

## Abbreviations

AIM: African index medicus
CHEC: Consensus on health economic criteria
DPT3: Diphtheria-pertussis-tetanus vaccine third dose
ICER: Incremental cost-effectiveness ratio
LILACS: Literatura Latino-Americana e do Caribe em Ciências da Saúde
LMIC: Low- and middle-income country
SSRN: Social Science Research Network
USD: United States dollar
WHO: World Health Organization
