# Supplementary material for: Systematic review of the costs and effectiveness of interventions to increase infant vaccination coverage in low- and middle-income countries
Source: BMC Health Serv Res. 2019 Oct 22;19:741. doi: 10.1186/s12913-019-4468-4 (PMC6806517; doi:10.1186/s12913-019-4468-4)
Supplement: Supplementary file 1 — Search terms for each database. (DOCX 16 kb) [file 12913_2019_4468_MOESM1_ESM.docx]

**Additional file 1: Search terms for each database.**

| **Database** | **Search terms** |
| --- | --- |
| PubMed | (coverage[Title/Abstract] OR uptake[Title/Abstract]) AND (immunization[Title/Abstract] OR immunisation[Title/Abstract] OR vaccination[Title/Abstract] OR vaccin*[Title/Abstract] OR EPI[Title/Abstract] OR immunization[MeSH Term] OR vaccination[MeSH Term] OR vaccines[MeSH Term] OR immunization programs*[MeSH Term]) AND (cost[Title/Abstract] OR costs[Title/Abstract] OR costing[Title/Abstract] OR cost-effective*[Title/Abstract] OR economic*[Title/Abstract] OR finance[Title/Abstract] OR cost benefit analys*[Title/Abstract] OR "costs and cost analysis"[MeSH Term] OR economics[MeSH Term] OR efficiency[MeSH Term] OR vaccination/economic*[ MeSH Term] OR Preventative Health Services/economic*[ MeSH Term] OR Communicable disease control/economic*[ MeSH Term] OR Health Promotion/economic*[ MeSH Term] OR health care costs[MeSH Term] OR cost-benefit analysis[MeSH Term]) AND (LMIC terms) |
| Cochrane Library | (coverage[Title/Abstract] OR uptake[Title/Abstract]) AND (immunization[Title/Abstract] OR immunisation[Title/Abstract] OR vaccination[Title/Abstract] OR vaccin*[Title/Abstract] OR EPI[Title/Abstract]) AND (cost[Title/Abstract] OR cost-effective*[Title/Abstract] OR economic[Title/Abstract] OR finance[Title/Abstract] OR cost benefit analysis[Title/Abstract]) AND (LMIC terms) |
| Embase | (coverage:ti,ab OR uptake:ti,ab) AND (immunization:ti,ab OR immunization:ti,ab OR vaccin*:ti,ab OR EPI:ti,ab) AND (cost*:ti,ab OR cost effective:ti,ab OR economic*:ti,ab OR finance:ti,ab OR cost benefit analys*:ti,ab) AND (LMIC terms) |
| Web of Science | (TI=(coverage OR uptake)) AND (TI=(immunization OR immunization OR vaccin* OR EPI)) AND (TI=(cost* OR cost effective OR economic* OR finance OR cost benefit analys* OR health care cost*)) AND (LMIC terms) |
| CEA Registry* | Immunization OR Immunisation OR Vaccine OR Vaccination |
| EconLit | (TI,AB(coverage) OR TI,AB(uptake)) AND (TI,AB(immunization) OR TI,AB(immunization) OR TI,AB(vaccination) OR TI,AB(vaccin*) OR TI,AB(EPI)) AND (TI,AB(cost*) OR TI,AB(cost effective) OR TI,AB(cost-effective) OR TI,AB(economic*) OR TI,AB(finance ) OR TI,AB(cost benefit analys*)) AND (LMIC terms) |
| AIM* | 3 title searches, Boolean expression: immunization, vaccine, cost |
| ELDIS* | immunization cost coverage |
| GreyNet* | immunization |
| Grey Literature Report* | Immunization (title) |
| World Bank Working Papers* | Immunization OR vaccine (title) |
| LILACS* | ti:(costo OR costos OR cost OR costs OR custo OR custos OR vacina OR vacuna OR inmunización OR imunização) AND (instance:"regional") AND ( db:("LILACS") AND limit:("humans") |
| LMIC terms | ("Afghanistan"[tiab] OR "Albania"[tiab] OR "Algeria"[tiab] OR "Samoa"[tiab] OR "Angola"[tiab] OR "Argentina"[tiab] OR "Armenia"[tiab] OR "Azerbaijan"[tiab] OR "Bangladesh"[tiab] OR "Belarus"[tiab] OR "Belize"[tiab] OR "Benin"[tiab] OR "Bhutan"[tiab] OR "Bolivia"[tiab] OR "Bosnia"[tiab] OR "Botswana"[tiab] OR "Brazil"[tiab] OR "Bulgaria"[tiab] OR "Burkina Faso"[tiab] OR "Burundi"[tiab] OR "Cabo Verde"[tiab] OR "Cape Verde"[tiab] OR "Cambodia"[tiab] OR "Cameroon"[tiab] OR "Central African Republic"[tiab] OR "CAR"[tiab] OR "Chad"[tiab] OR "China"[tiab] OR "Colombia"[tiab] OR "Comoros"[tiab] OR "DRC"[tiab] OR "Congo"[tiab] OR "Zaire"[tiab] OR "Costa Rica"[tiab] OR "Cote d'Ivoire"[tiab] OR "Ivory Coast"[tiab] OR "Cuba"[tiab] OR "Djibouti"[tiab] OR "Dominica"[tiab] OR "Dominican Republic"[tiab] OR "Ecuador"[tiab] OR "Egypt"[tiab] OR "El Salvador"[tiab] OR "Equatorial Guinea"[tiab] OR "Eritrea"[tiab] OR "Ethiopia"[tiab] OR "Fiji"[tiab] OR "Gabon"[tiab] OR "Gambia"[tiab] OR "Georgia"[tiab] OR "Ghana"[tiab] OR "Grenada"[tiab] OR "Guatemala"[tiab] OR "Guinea"[tiab] OR "Guinea-Bissau"[tiab] OR "Guyana"[tiab] OR "Haiti"[tiab] OR "Honduras"[tiab] OR "India"[tiab] OR "Indonesia"[tiab] OR "Iran"[tiab] OR "Iraq"[tiab] OR "Jamaica"[tiab] OR "Jordan"[tiab] OR "Kazakhstan"[tiab] OR "Kenya"[tiab] OR "Kiribati"[tiab] OR "Korea"[tiab] OR "Kosovo"[tiab] OR "Kyrgyz"[tiab] OR "Kyrgyzstan"[tiab] OR "Lao"[tiab] OR "Laos"[tiab] OR "Lebanon"[tiab] OR "Lesotho"[tiab] OR "Liberia"[tiab] OR "Libya"[tiab] OR "Macedonia"[tiab] OR "Madagascar"[tiab] OR "Malawi"[tiab] OR "Malaysia"[tiab] OR "Maldives"[tiab] OR "Mali"[tiab] OR "Marshall"[tiab] OR "Mauritania"[tiab] OR "Mauritius"[tiab] OR "Mexico"[tiab] OR "Micronesia"[tiab] OR "Moldova"[tiab] OR "Mongolia"[tiab] OR "Montenegro"[tiab] OR "Morocco"[tiab] OR "Mozambique"[tiab] OR "Myanmar"[tiab] OR "Burma"[tiab] OR "Namibia"[tiab] OR "Nepal"[tiab] OR "Nicaragua"[tiab] OR "Niger"[tiab] OR "Nigeria"[tiab] OR "Pakistan"[tiab] OR "Palau"[tiab] OR "Panama"[tiab] OR "Papua New Guinea"[tiab] OR "Paraguay"[tiab] OR "Peru"[tiab] OR "Philippines"[tiab] OR "Romania"[tiab] OR "Russian Federation"[tiab] OR "Russia"[tiab] OR "Rwanda"[tiab] OR "Samoa"[tiab] OR "Sao Tome and Principe"[tiab] OR "Principe"[tiab] OR "Senegal"[tiab] OR "Serbia"[tiab] OR "Sierra Leone"[tiab] OR "Solomon Islands"[tiab] OR "Somalia"[tiab] OR "South Africa"[tiab] OR "South Sudan"[tiab] OR "Sri Lanka"[tiab] OR "St. Lucia"[tiab] OR "St. Vincent and the Grenadines"[tiab] OR "Vincent"[tiab] OR "Sudan"[tiab] OR "Suriname"[tiab] OR "Swaziland"[tiab] OR "Syrian Arab Republic"[tiab] OR "Syria"[tiab] OR "Tajikistan"[tiab] OR "Tanzania"[tiab] OR "Thailand"[tiab] OR "Timor-Leste"[tiab] OR "Timor Leste"[tiab] OR "Togo"[tiab] OR "Tonga"[tiab] OR "Tunisia"[tiab] OR "Turkey"[tiab] OR "Turkmenistan"[tiab] OR "Tuvalu"[tiab] OR "Uganda"[tiab] OR "Ukraine"[tiab] OR "Uzbekistan"[tiab] OR "Vanuatu"[tiab] OR "Venezuela"[tiab] OR "Vietnam"[tiab] OR "Viet Nam"[tiab] OR "West Bank"[tiab] OR "Gaza"[tiab] OR "Yemen"[tiab] OR "Zambia"[tiab] OR "Zimbabwe"[tiab] OR "Rhodesia"[tiab] OR "Africa"[tiab] OR "Africa"[Mesh] OR "Central America"[Mesh] OR "Argentina"[Mesh] OR "Bolivia"[Mesh] OR "Brazil"[Mesh] OR "Colombia"[Mesh] OR "Ecuador"[Mesh] OR "Guyana"[Mesh] OR "Paraguay"[Mesh] OR "Peru"[Mesh] OR "Suriname"[Mesh] OR "Venezuela"[Mesh] OR "Mexico"[Mesh] OR "Asia, Central"[Mesh] OR "Russia"[Mesh] OR "Cambodia"[Mesh] OR "Timor-Leste"[Mesh] OR "Indonesia"[Mesh] OR "Laos"[Mesh] OR "Malaysia"[Mesh] OR "Mekong Valley"[Mesh] OR "Myanmar"[Mesh] OR "Philippines"[Mesh] OR "Thailand"[Mesh] OR "Vietnam"[Mesh] OR "Bangladesh"[Mesh] OR "India"[Mesh] OR "Afghanistan"[Mesh] OR "Iran"[Mesh] OR "Iraq"[Mesh] OR "Jordan"[Mesh] OR "Lebanon"[Mesh] OR "Syria"[Mesh] OR "Turkey"[Mesh] OR "Yemen"[Mesh] OR "Nepal"[Mesh] OR "Pakistan"[Mesh] OR "Sri Lanka"[Mesh] OR "China"[Mesh] OR "Mongolia"[Mesh] OR "Albania"[Mesh] OR "Bosnia and Herzegovina"[Mesh] OR "Bulgaria"[Mesh] OR "Kosovo"[Mesh] OR "Macedonia (Republic)"[Mesh] OR "Moldova"[Mesh] OR "Montenegro"[Mesh] OR "Republic of Belarus"[Mesh] OR "Romania"[Mesh] OR "Russia"[Mesh] OR "Serbia"[Mesh] OR "Developing countries"[Mesh]) |

*Database search limitations prevented low- and middle-income country (LMIC) terms from being included in these databases.
