# Supplementary material for: Systematic review of the costs and effectiveness of interventions to increase infant vaccination coverage in low- and middle-income countries
Source: BMC Health Serv Res. 2019 Oct 22;19:741. doi: 10.1186/s12913-019-4468-4 (PMC6806517; doi:10.1186/s12913-019-4468-4)
Supplement: Supplementary file 2 — Consensus on Health Economic Criteria (CHEC) list for health economic evaluations35 and study scores. (DOCX 19 kb) [file 12913_2019_4468_MOESM2_ESM.docx]

Additional file 2: Consensus on Health Economic Criteria (CHEC) list for health economic evaluations^35^ and study scores.

| **Item** | **Hayford 2014** | **Banerjee 2010** | **Khan 2013** | **Powell-Jackson 2018** | **Byberg 2017** | **Drain 2003** | **Andersson 2009** | **Levin 2005** | **Owais 2011** | **Pandey 2007** | **Barham 2007** | **Carnell 2014** | **Rainey 2009** | **Soeung 2006** |
| --- | --- | --- | --- | --- | --- | --- | --- | --- | --- | --- | --- | --- | --- | --- |
| 1. Is the study population clearly described? | 1 | 1 | 1 | 1 | 1 | 1 | 1 | 0 | 1 | 1 | 1 | 1 | 1 | 1 |
| 2. Are competing alternatives clearly described? | 1 | 1 | 1 | 1 | 1 | 1 | 1 | 1 | 1 | 1 | 1 | 1 | 1 | 1 |
| 3. Is a well-defined research question posed in answerable form? | 1 | 1 | 1 | 1 | 1 | 1 | 1 | 1 | 1 | 1 | 1 | 1 | 1 | 1 |
| 4. Is the economic study design appropriate to the stated objective? | 1 | 1 | 1 | 1 | 1 | 1 | 1 | 1 | 1 | 1 | 1 | 1 | 1 | 1 |
| 5. Is the chosen time horizon appropriate to include relevant costs and consequences? | 1 | 1 | 1 | 1 | 1 | 1 | 1 | 1 | 1 | 1 | 1 | 1 | 1 | 1 |
| 6. Is the actual perspective chosen appropriate? | 1 | 1 | 1 | 1 | 1 | 1 | 1 | 1 | 1 | 1 | 1 | 1 | 1 | 1 |
| 7. Are all important and relevant costs for each alternative identified? | 1 | 0 | 1 | 0 | 1 | 0 | 0 | 0 | 0 | 0 | 0 | 0 | 0 | 0 |
| 8. Are all costs measured appropriately in physical units? | 1 | 1 | 1 | 1 | 1 | 1 | 1 | 1 | 1 | 1 | 1 | 1 | 1 | 1 |
| 9. Are costs valued appropriately? | 1 | 1 | 1 | 1 | 0 | 1 | 0 | 1 | 1 | 1 | 1 | 0 | 1 | 1 |
| 10. Are all important and relevant outcomes for each alternative identified? | 1 | 1 | 1 | 1 | 1 | 1 | 1 | 0 | 0 | 0 | 0 | 0 | 0 | 0 |
| 11. Are all outcomes measured appropriately? | 1 | 1 | 1 | 1 | 1 | 1 | 1 | 1 | 1 | 1 | 1 | 1 | 1 | 1 |
| 12. Are outcomes valued appropriately? | 1 | 1 | 1 | 1 | 1 | 1 | 1 | 1 | 1 | 1 | 1 | 1 | 1 | 1 |
| 13. Is an incremental analysis of costs and outcomes of alternatives performed? | 1 | 1 | 0 | 1 | 1 | 1 | 0 | 1 | 0 | 0 | 0 | 0 | 0 | 0 |
| 14. Are all future costs and outcomes discounted appropriately?* | NA | NA | NA | NA | NA | NA | NA | NA | NA | NA | NA | NA | NA | NA |
| 15. Are all important variables, whose values are uncertain, appropriately subjected to sensitivity analysis?* | NA | NA | NA | NA | NA | NA | NA | NA | NA | NA | NA | NA | NA | NA |
| 16. Do the conclusions follow from the data reported? | 1 | 1 | 1 | 1 | 1 | 1 | 1 | 1 | 1 | 1 | 1 | 1 | 1 | 1 |
| 17. Does the study discuss the generalizability of the results to other settings and patient/client groups? | 1 | 1 | 1 | 1 | 0 | 0 | 0 | 1 | 0 | 0 | 0 | 0 | 0 | 1 |
| 18. Does the article indicate that there is no potential conflict of interest of study researcher(s) and funder(s)? | 1 | 1 | 1 | 1 | 1 | 1 | 1 | 1 | 1 | 1 | 1 | 1 | 1 | 0 |
| 19. Are ethical and distributional issues discussed appropriately? | 1 | 1 | 1 | 1 | 1 | 0 | 1 | 0 | 1 | 1 | 0 | 1 | 0 | 0 |
| **Total CHEC-list Score (out of 17)** | **17** | **16** | **16** | **16** | **15** | **14** | **13** | **13** | **13** | **13** | **12** | **12** | **12** | **12** |

*This item was excluded as specific to modeling analyses, which were not included in our review.
